# Diagnostic challenges in COVID 19 and dengue co-infection: a case series report from tertiary care centers of Saudi Arabia

**DOI:** 10.3389/fmed.2026.1861511

**Published:** 2026-06-10

**Authors:** Ashwaq M. Al-Nazawi, Maseer Khan, Anjum Qadir, Rana Alghamdi, Alzahrani Ali Ahmed, Wafa Ali Hetany, Mohammad H. Alharbi

**Affiliations:** 1 Department of Public Health, College of Nursing and Health Sciences, Jazan University, Jazan, Saudi Arabia; 2 Laboratory Department, Jazan University Hospital, Jazan University, Jazan, Saudi Arabia; 3 Health Holding Company, Jeddah, Saudi Arabia; 4 Vector-Borne & Zoonotic Diseases Administration, Public Health, Second Health Cluster, Jeddah, Saudi Arabia; 5 Preventive Medicine Department, Public Health Directorate, Ministry of Health, Jeddah, Saudi Arabia

**Keywords:** case series, co-infection, COVID-19, dengue, Saudi Arabia

## Abstract

**Background:**

COVID-19 has changed the pattern of clinical diagnosis and laboratory confirmation for many communicable diseases making it essential for treatment procedures overburdening the health care system. Dengue and COVID-19, though caused by distinct viral pathogens, often present with overlapping clinical features, particularly in tropical and endemic regions. This overlap poses significant diagnostic challenges, especially during co-epidemics.

**Objective:**

To analyze the clinical presentation of patients suspected of dengue and/or COVID-19, and to highlight key similarities and differences that may assist in timely diagnosis and management.

**Methods:**

A retrospective case series of 50 patients admitted to multiple hospitals in a city in Saudi Arabia during a period of concurrent dengue and COVID-19 transmission was conducted. Patients were evaluated based on demographic characteristics, presenting symptoms, laboratory findings, and final diagnoses.

**Results:**

The mean age of patients was 39 ± 11.8 years, with 86% male and 14% female participants. Clinical overlap was considerable—fever, headache, myalgia, and rash were commonly reported in both infections. Notably, a cutaneous rash, classically associated with dengue, was observed in 36% of patients, including some confirmed COVID-19 cases. Misdiagnoses occurred due to atypical presentations, including a case initially diagnosed as dengue based on a petechial rash, later confirmed to be COVID-19. Co-infection cases exhibited more severe symptomatology and prolonged recovery. Most patients recovered after supportive management. However, co-infected patients demonstrated extended hospitalization and increased disease severity compared with mono-infected cases.

**Conclusion:**

This case series underscores the diagnostic complexity posed by the clinical similarity between dengue and COVID-19, particularly in endemic settings. Accurate and early differentiation through combined clinical and laboratory approaches is crucial to avoid misdiagnosis, ensure appropriate treatment, and reduce morbidity.

## 1 Introduction

While the world stands for the resurgence of another COVID-19 wave, evidenced by the identification of 228 cases within a single week across diverse geographic locations (1), dengue-endemic regions face a growing public health challenge. The COVID-19 pandemic era has seen tremendous increase in dengue cases (2). As the world struggled with COVID-19 pandemic, dengue endemic regions posed double pandemic (3). The overlapping clinical manifestations of COVID-19 and dengue fever have engendered diagnostic ambiguity (4), complicating case management, exacerbating disease severity, and imposing an augmented strain on already overburdened healthcare infrastructures (5). As per the latest WHO statistical reports Saudi Arabia witnessed 28 deaths of COVID-19 in a week (1). The similarity in case identification in tropical regions with arbovirus infections (6) make diagnosis difficult and mandate testing for both COVID-19 and dengue confirming through RTPCR, Dengue IgM, Dengue NS1 antigen (7, 8).

Dengue, a leading and most common (9) arboviral infection is caused by Flavivirus with in Flaviviridae family transmitted by mosquito *Aedes aegypti*. Saudi Arabia being a pilgrimage receive devotees from all over the world, and it will increase the risk of dengue importation creating a public health problem (10, 25). Dengue is the most significant arboviral disease responsible for approximately 10,000 deaths and 100 million symptomatic cases annually across more than 125 countries. Nearly 50% of the world’s population resides in regions where environmental conditions favor dengue transmission (10). More than 6 billion will be in dengue endemic areas by 2080 according to future projections (11). Dengue is caused specifically by 4 serotypes DENV-1, DENV-2, DENV-3, DENV-4 (12) and the 5th serotype identified DENV-5 (13).

The global community experienced unprecedented disruption and devastation due to the COVID-19 pandemic during the years 2021 and 2022. Globally approximately 16 million people died from the COVID-19 pandemic in 2020 and 2021. This reduced global life expectancy by 1.6 years (14). Additionally, mortality among individuals aged 15 years and older increased by 22% for males and 17% for females (14). The COVID-19 pandemic has highlighted the importance of population studies in explaining the disease injury and burden (14). Along with causing extensive mortality, COVID-19 increased vulnerability for diseases by impairing immune system causing increased susceptibility for infections (15, 16). Dengue and COVID-19 co-infection challenged the advanced medical technology with similar laboratory and clinical features (6, 7, 26). Several mortality cases reported in varied areas with these co infections (3, 17, 18). Dengue infections increase while COVID-19 leave the patient susceptible with reduced immunity (19). There is very limited literature available on dengue and COVID-19 co-infections that highlights the comparison of symptoms and diagnosis based on clinical features. In this context, the current article presents a case series that highlights the clinical similarities in the presentation of dengue and COVID-19, emphasizing the diagnostic challenges posed by overlapping symptoms.

Aim of the study: This study aims to analyze and compare the clinical presentation of dengue and COVID-19 in co-infected patients, with a focus on the implications for accurate diagnosis and effective case management.

## 2 Methodology

### 2.1 Study design

**Figure 1:**
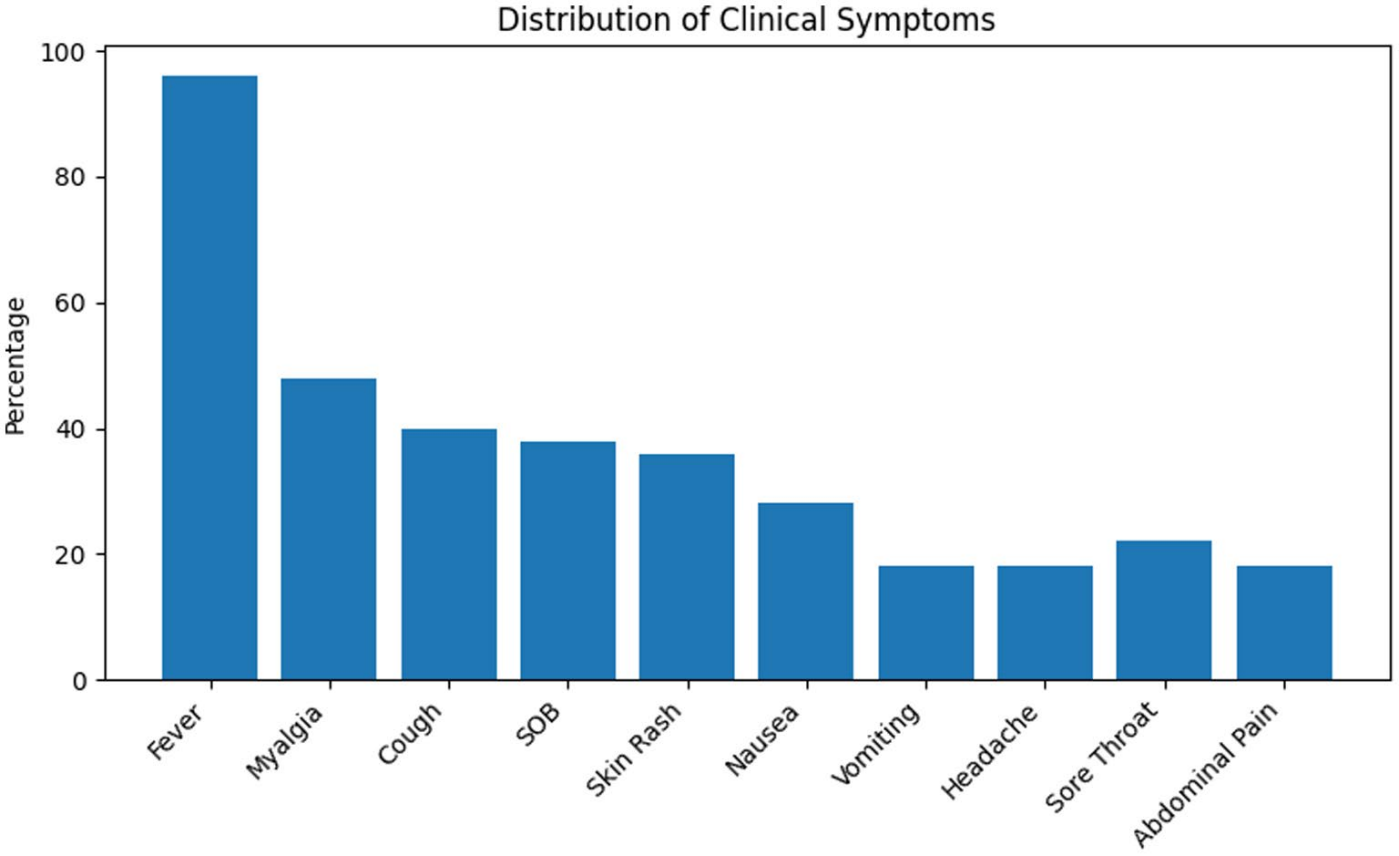
The percentage distribution of major clinical symptoms among study participants.

**Figure 2:**
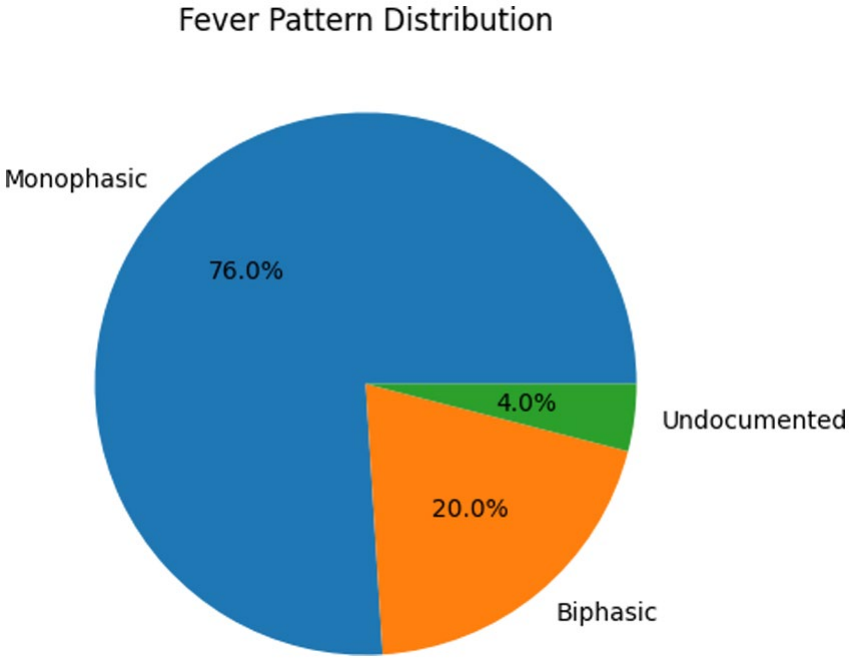
The distribution of monophasic and biphasic fever patterns observed among patients.

This is a retrospective, descriptive case series conducted at tertiary care hospitals in Jeddah, Saudi Arabia. We reviewed clinical and laboratory records of 50 patients admitted between March and June 2020 with confirmed or suspected COVID-19, dengue, or co-infection. Cases were included if laboratory confirmation was available by RT-PCR for SARS-CoV-2 and/or NS1 antigen, IgM/IgG serology, or PCR for dengue. Demographic, clinical, and outcome data were extracted from patient records and analyzed descriptively Manuscript Formatting. Suspected COVID-19 cases referred to patients presenting with compatible clinical symptoms and epidemiological exposure before RT-PCR confirmation (Figures 1, 2).

### 2.2 Inclusion criteria

1. RT-PCR confirmed cases of COVID-19.
2. Dengue confirmation through NS1 antigen, IgM/IgG serology, or PCR.
3. Availability of detailed clinical symptoms and demographic information.

Data extracted from patient case records and surveillance systems. Each patient’s file was reviewed for demographics (age, gender, nationality), clinical symptoms (including fever pattern, myalgia, respiratory and gastrointestinal features), and outcomes (hospitalization, severity, and death). Cases were categorized into monophasic and biphasic fever patterns based on clinical notes and temperature chart trends. Descriptive statistics were used to summarize categorical data, and symptom frequencies were analyzed. Inferential statistical analyses including chi-square testing were additionally performed where applicable to compare symptom distribution among infection categories, with *p* < 0.05 considered statistically significant (Table 1).

**Table 1:** Demographic characteristics and clinical symptom profile of patients with suspected dengue and COVID-19.

Total patients	No. of cases	% of cases
Male	43	86%
Female	7	14%
Saudi	23	46%
Non Saudi	27	54%
Freq. automatic calc.		
Cough	20	40%
Fever	48	96%
SOB	19	38%
Vomiting	9	18%
Headache	9	18%
Body ache (myalgia)	24	48%
Joint pain (arthralgia)	2	4%
Sore throat	11	22%
Nausea	14	28%
Skin rash	18	36%
Dry cough	8	16%
Productive cough	12	24%
Abdominal pain	9	18%
Age		
Mean age	39 ± 11.8	

### 2.3 Exclusion criteria

1. Patients with incomplete clinical or laboratory records.
2. Patients without confirmatory laboratory testing for dengue or COVID-19.
3. Patients diagnosed with other confirmed febrile illnesses unrelated to dengue or COVID-19.

## 3 Results

This case series report is of 50 patients admitted in various hospitals for the symptoms of dengue/COVID-19/co-infections. Mean age of the patients found to be 39 ± 11.8 years, we observed considerable overlap in symptom profiles, contributing to diagnostic complexity.

Fever was present in all patients, with biphasic fever reported in 20% of cases (*n* = 10), a feature more typical of dengue, whereas monophasic fever was observed in 76% (*n* = 38), a feature of COVID-19 or other viral fevers. 4% (*n* = 2) had undocumented fever patterns. This distinction may reflect temporal overlap in symptom evolution or the limitations of patient recall and documentation. Graphical representations of monophasic and biphasic fever curves were added to better demonstrate the observed temperature patterns (Table 2).

**Table 2:** Inferential and descriptive statistical analysis of overlapping clinical manifestations in dengue and COVID-19.

Statistical analysis	Variables compared	Statistical test	Result	*p*-value	Interpretation
Symptom association analysis	Respiratory symptoms vs. gastrointestinal symptoms	Fisher’s exact test	Significant association observed	8.47 × 10^−8^	Patients presenting with respiratory symptoms were significantly more likely to have gastrointestinal symptoms simultaneously
Fever pattern distribution	Monophasic vs. biphasic fever	Descriptive analysis	Monophasic fever more common	—	Fever pattern alone was not sufficient for differentiating infections
Respiratory symptom frequency	SOB, dry cough, productive cough, sore throat	Frequency analysis	Respiratory manifestations common	—	Supports overlap with COVID-19 clinical presentation
Gastrointestinal symptom frequency	Nausea, vomiting, abdominal pain	Frequency analysis	GI symptoms frequently observed	—	Demonstrates overlap between dengue and COVID-19 manifestations
Musculoskeletal symptoms	Myalgia and arthralgia	Frequency analysis	Myalgia common, arthralgia rare	—	Musculoskeletal symptoms had limited discriminatory value
Dermatological manifestations	Skin rash	Frequency analysis	Rash present in 36% cases	—	Rash alone may mislead diagnosis toward dengue

Myalgia was the most frequently reported musculoskeletal symptom, present in 46% of cases. Arthralgia, a classical symptom of dengue, was surprisingly infrequent, noted in only 4%. Headache occurred in 18% of patients. These findings suggest that while musculoskeletal symptoms remain important, their diagnostic utility may be limited in co-infection settings.

Respiratory symptoms—which are more indicative of COVID-19—were prominent: shortness of breath (38%), productive cough (24%), and dry cough (14%). These manifestations complicate the clinical picture, especially in the presence of overlapping systemic symptoms.

Cutaneous rash, commonly seen in dengue, was reported in 36% of patients, while gastrointestinal symptoms such as nausea (28%), vomiting (18%), and abdominal pain (18%) were also frequent, reflecting the known overlap of dengue and COVID-19 in affecting the gastrointestinal tract. Sore throat was present in only 20% of patients, underscoring its limited discriminatory value in co-infected cases.

These findings emphasize the diagnostic ambiguity posed by co-infection. The concurrent presence of respiratory and systemic symptoms can easily mask or mimic either illness, delaying appropriate care and the study highlights the urgent need for dual diagnostic testing, integrated clinical algorithms, and syndromic surveillance strategies in regions experiencing overlapping outbreaks.

## 4 Discussion

This case series reporting shows the importance of knowledge regarding COVID-19 and Dengue co-infections highlighting the complex clinical interplay between COVID-19 and dengue fever, putting forward diagnostic challenges in dengue endemic regions (1) like coastal areas of Saudi Arabia. Among the 50 patients studied, the symptom overlapping between dengue, COVID-19, and their co-infections blurred conventional diagnostic pathways, stressing the need for increased clinical understanding and the necessity of integrated diagnostic strategies.

### 4.1 Demographic overview

The mean age of patients in this series was 39 ± 11.8 years, with a strong male predominance (86%). This gender imbalance could reflect differential health-seeking behaviour and occupational exposure risks of the population under study. The slight predominance of non-Saudi patients (54%) also suggests potential disparities in exposure or healthcare access, particularly among migrant populations who may live in densely populated housing or have limited access to preventive measures.

### 4.2 Fever patterns and diagnostic implications

Fever was the most consistent symptom observed in 96% of cases. Importantly, 20% of patients exhibited a biphasic fever, a pattern more characteristic of dengue virus infection. Monophasic fever, seen in 76% of cases, is typically associated with COVID-19 and other viral illnesses. The small percentage (4%) of patients (Supplementary Table 1) with undocumented fever patterns reflects possible limitations in retrospective data collection or patient recall. The variability in fever presentation underscores the need for clinicians to consider both temporal and qualitative aspects of fever when differentiating between the two infections. This shows based on fever patterns it is difficult to diagnose dengue and COVID-19.

### 4.3 Musculoskeletal symptoms: limited discriminatory value

Myalgia, reported in 48% of cases, emerged as the most common musculoskeletal complaint, whereas arthralgia—a hallmark symptom of dengue—was surprisingly rare (4%) (Supplementary Table 1). The triad of symptoms fever, myalgia and arthralgia would always direct physician towards dengue in the pre COVID era, however post COVID-19 season, it is not surprising to have these symptoms in co-infection. This pose clinicians diagnostic challenge unless laboratory confirmation is followed. The same view was expressed in the meta-analysis done by Dalugama and Seneviratne (4). These findings suggest that musculoskeletal symptoms alone, while commonly seen, may offer limited specificity in distinguishing between the two diseases in overlapping outbreaks. Headache occurred in 18% of patients. In the study done by Carosella et al. (20), headaches are seen more commonly in coinfected patients.

### 4.4 Respiratory manifestations: hallmark of COVID-19

As expected, respiratory symptoms were notably prevalent: shortness of breath (38%), productive cough (24%), and dry cough (16%). These findings align with known clinical features of COVID-19 and may indicate either isolated COVID-19 infections or the respiratory burden of co-infections. The coexistence of such symptoms with systemic features (e.g., fever, myalgia) complicates the clinical picture and emphasizes the need for respiratory assessment even in patients presenting with presumed dengue symptoms. Dengue hemorrhagic fever may cause pulmonary oedema and pleural effusion which may exacerbate shortness of breath. While COVID-19 causes primarily breathlessness by causing inflammation of alveoli or by pulmonary intravascular coagulation (21). Sore throat was observed in only 22% of patients, reaffirming its limited diagnostic utility in co-infection settings. While often cited as a typical symptom of upper respiratory viral infections, sore throat is less common in dengue and in COVID-19 lower respiratory tract infections are frequent (22).

### 4.5 Cutaneous and gastrointestinal manifestations

Cutaneous rash, typically associated with dengue, was reported in 36% of patients—substantially higher than commonly seen in COVID-19 cases. In a case reported in Thailand the patient initially presented with white petechial rash hinting clinicians towards dengue, however later it was diagnosed to be COVID-19 (23). This case stresses the pitfalls in relying solely on clinical presentation, particularly cutaneous findings, in settings where both dengue and COVID-19 are circulating. The overlapping symptoms—such as fever, rash, myalgia, thrombocytopenia, and elevated liver enzymes gastrointestinal symptoms, such as nausea (28%), vomiting (18%), and abdominal pain (18%), were also frequent. These manifestations reflect the overlapping systemic involvement of both viruses and further highlight the diagnostic uncertainty. The co-occurrence of skin and gastrointestinal symptoms should prompt consideration of dengue, but not to the exclusion of COVID-19, especially in co-circulation settings. The significant symptom overlaps can lead to misdiagnosis, delayed treatment, and inappropriate infection control measures. In febrile patients presenting with a mix of systemic, respiratory, and gastrointestinal symptoms, clinicians must maintain a high index of suspicion for co-infection, particularly in endemic or high-burden areas. Additional information regarding patient outcomes included in Supplementary Table 3.

The findings highlight the critical need for dual diagnostic testing for dengue and SARS-CoV-2 in febrile patients, integrated clinical algorithms that account for the co-prevalence of these diseases and Syndromic surveillance systems that can identify emerging patterns of co-infection. While laboratory confirmation has proved the coinfection of dengue and COVID-19, it is always required laboratory testing for Dengue NS1, Dengue PCR and RTPCR for COVID-19 together (Supplementary Table 2) when a patient arrives for fever. Integrated clinical algorithms need to be created stating clear demarcation between clinical symptoms. Keeping in mind the financial resources required for laboratory confirmation of all fever cases, it is always beneficial to create clinical algorithms which will delineate the cases to the best possible way by the clinicians there by reducing the burden on health care system and laboratory confirmation can be restricted to the critically ill patients (24).

## 5 Limitations and future directions

Although we have collected 50 samples still, we find this as a limitation of the study which may limit the generalizability of the findings. Additionally, not all cases may have had laboratory-confirmed diagnoses of both dengue and COVID-19, potentially introducing classification bias. Documentation variability and reliance on patient-reported symptoms also pose limitations.

Future studies should aim for larger, multicentric cohorts with confirmed dual infections. Investigations into the immunological interactions between dengue virus and SARS-CoV-2 may help explain atypical symptom presentations and disease severity. Additionally, research into potential biomarkers that can differentiate between dengue and COVID-19 could greatly enhance early diagnostic accuracy.

## 6 Conclusion

This study highlights the diagnostic challenges caused by overlapping manifestations of dengue and COVID-19 in endemic settings. Combined clinical assessment and laboratory confirmation remain essential for accurate diagnosis and timely management. Integrated diagnostic algorithms and dual testing strategies may help reduce misdiagnosis and improve patient outcomes during overlapping outbreaks.

## 7 Recommendations

Safeguarding dengue-endemic nations against the “double pandemic” threat will require a multilayered approach: sustained vector control, vaccination where available and integrated clinical algorithms for syndromic identification by clinicians. Implementing these integrated measures now may reduce burden on health care system.

## Data Availability

The original contributions presented in the study are included in the article/Supplementary material, further inquiries can be directed to the corresponding authors.

## References

[1] COVID-19 Data WHO COVID-19 dashboard. Available online at: https://data.who.int/dashboards/covid19/data (Accessed June 26, 2025)

[2] León-Figueroa DA Abanto-Urbano S Olarte-Durand M Nuñez-Lupaca JN Barboza JJ Bonilla-Aldana DK . COVID-19 and dengue coinfection in Latin America: a systematic review. New Microbes New Infect. (2022) 49-50:101041. doi: 10.1016/j.nmni.2022.101041, 36320316 PMC9613782

[3] Ridwan R (2020) COVID-19 and dengue: a deadly duo. Available online at: https://journals.sagepub.com/doi/abs/10.1177/0049475520936874 (Accessed July 9, 2025) 10.1177/0049475520936874 32588763

[4] Dalugama C Seneviratne SL. Dengue and COVID-19 co-infections: an important consideration in the tropics. Trans R Soc Trop Med Hyg. (2023) 117:241–54. doi: 10.1093/trstmh/trac114, 36479900

[5] Kumar S Mishra A. A narrative review on dengue and COVID-19 co-infection: a deadly duo. J Med Evid. (2024) 5:55. doi: 10.4103/JME.JME_57_23

[6] Al-Nazawi AM Al-Zahrani AA Qadir A Alghamdi R Tambo E Alsahafi A. Case report: A fatal outcome from co-infection of COVID-19 and dengue in the western region of Jeddah, Saudi Arabia. Front Public Health. (2022) 10:942381. doi: 10.3389/fpubh.2022.942381, 36051997 PMC9424996

[7] Khalil A Badr B Wright H Talo M Atteiya M. Dengue fever and COVID-19 co-infection at the emergency Department of a Tertiary Care Hospital in Jeddah, Saudi Arabia. Eur J Case Rep Intern Med. (2020) 7:002167. doi: 10.12890/2020_002167, 33457380 PMC7806309

[8] Chen N Zhou M Dong X Qu J Gong F Han Y . Epidemiological and clinical characteristics of 99 cases of 2019 novel coronavirus pneumonia in Wuhan, China: a descriptive study. Lancet. (2020) 395:507–13. doi: 10.1016/S0140-6736(20)30211-7, 32007143 PMC7135076

[9] Raafat N Blacksell SD Maude RJ. A review of dengue diagnostics and implications for surveillance and control. Trans R Soc Trop Med Hyg. (2019) 113:653–60. doi: 10.1093/trstmh/trz068, 31365115 PMC6836713

[10] Alharbi AM. The increasing importance of dengue virus infection in Saudi Arabia: A review. Virus Res. (2025) 351:199510. doi: 10.1016/j.virusres.2024.199510, 39681278 PMC11732239

[11] Messina JP Brady OJ Golding N Kraemer MUG Wint GRW Ray SE . The current and future global distribution and population at risk of dengue. Nat Microbiol. (2019) 4:1508–15. doi: 10.1038/s41564-019-0476-8, 31182801 PMC6784886

[12] Lee MF Wu YS Poh CL. Molecular mechanisms of antiviral agents against dengue virus. Viruses. (2023) 15:705. doi: 10.3390/v15030705, 36992414 PMC10056858

[13] Lessa CLS Hodel KVS Gonçalves M d S Machado BAS. Dengue as a disease threatening global health: a narrative review focusing on Latin America and Brazil. Trop Med Infect Dis. (2023) 8:5. doi: 10.3390/tropicalmed8050241, 37235289 PMC10221906

[14] Schumacher AE Kyu HH Aali A Abbafati C Abbas J Abbasgholizadeh R . Global age-sex-specific mortality, life expectancy, and population estimates in 204 countries and territories and 811 subnational locations, 1950–2021, and the impact of the COVID-19 pandemic: a comprehensive demographic analysis for the global burden of disease study 2021. Lancet. (2024) 403:1989–2056. doi: 10.1016/S0140-6736(24)00476-8, 38484753 PMC11126395

[15] Chen C Zhou W Cui Y Cao K Chen M Qu R . Global, regional, and national characteristics of the main causes of increased disease burden due to the covid-19 pandemic: time-series modelling analysis of global burden of disease study 2021. BMJ. (2025) 390:e083868. doi: 10.1136/bmj-2024-083868, 40602809 PMC12216812

[16] Krumbein H Kümmel LS Fragkou PC Thölken C Hünerbein BL Reiter R . Respiratory viral co-infections in patients with COVID-19 and associated outcomes: a systematic review and meta-analysis. Rev Med Virol. (2023) 33:e2365. doi: 10.1002/rmv.2365, 35686619 PMC9347814

[17] Butt MH Ahmad A Misbah S Mallhi TH Khan YH. Dengue fever and COVID-19 coinfection; a threat to public health for coepidemic in Pakistan. J Med Virol. (2021) 93:671–2. doi: 10.1002/jmv.26464, 32852782 PMC7461537

[18] Saavedra-Velasco M Chiara-Chilet C Pichardo-Rodriguez R Grandez-Urbina A Inga-Berrospi F. Coinfección entre dengue y COVID-19: Necesidad de abordaje en zonas endémicas. Rev Fac Cien Med Univ Nac Cordoba. (2020) 77:52–54. Available online at: https://revistas.unc.edu.ar/index.php/med/article/view/28031 (Accessed June 26, 2025). 32238260 10.31053/1853.0605.v77.n1.28031

[19] El-Qushayri AE Kamel AMA Reda A Ghozy S. Does dengue and COVID-19 co-infection have worse outcomes? A systematic review of current evidence. Rev Med Virol. (2022) 32:e2339. doi: 10.1002/rmv.2339, 35213764 PMC9111070

[20] Carosella LM Pryluka D Maranzana A Barcan L Cuini R Freuler C . Characteristics of patients co-infected with severe acute respiratory syndrome coronavirus 2 and dengue virus, Buenos Aires, Argentina, march–June 2020. Emerg Infect Dis. (2021) 27:348–51. doi: 10.3201/eid2702.203439, 33347804 PMC7853556

[21] McGonagle D O’Donnell JS Sharif K Emery P Bridgewood C. Immune mechanisms of pulmonary intravascular coagulopathy in COVID-19 pneumonia. Lancet Rheumatol. (2020) 2:e437–45. doi: 10.1016/S2665-9913(20)30121-1, 32835247 PMC7252093

[22] Nicholls JM Poon LLM Lee KC Ng WF Lai ST Leung CY . Lung pathology of fatal severe acute respiratory syndrome. Lancet. (2003) 361:1773–8. doi: 10.1016/S0140-6736(03)13413-7, 12781536 PMC7112492

[23] Joob B Wiwanitkit V. COVID-19 can present with a rash and be mistaken for dengue. J Am Acad Dermatol. (2020) 82:e177. doi: 10.1016/j.jaad.2020.03.036, 32213305 PMC7156802

[24] Saddique A Rana MS Alam MM Ikram A Usman M Salman M . Emergence of co-infection of COVID-19 and dengue: A serious public health threat. J Infect. (2020) 81:e16–8. doi: 10.1016/j.jinf.2020.08.009, 32800797 PMC7422859

[25] Al-Nefaie H Alsultan A Abusaris R. Temporal and spatial patterns of dengue geographical distribution in Jeddah, Saudi Arabia. J Infect Public Health. (2022) 15:1025–35. doi: 10.1016/j.jiph.2022.08.003, 36007387

[26] Wu D Lu J Liu Q Ma X He W. To alert coinfection of COVID-19 and dengue virus in developing countries in the dengue-endemic area. Infect Control Hosp Epidemiol. (2020) 41:1482–2. doi: 10.1017/ice.2020.187, 32362302 PMC7218187

